# Genetic Diversity and Identification of Homozygosity-Rich Genomic Regions in Seven Italian Heritage Turkey (*Meleagris gallopavo*) Breeds

**DOI:** 10.3390/genes12091342

**Published:** 2021-08-28

**Authors:** Francesca Bernini, Alessandro Bagnato, Stefano Paolo Marelli, Luisa Zaniboni, Silvia Cerolini, Maria Giuseppina Strillacci

**Affiliations:** Department of Veterinary Medicine, Università degli Studi di Milano, Via dell’Università 6, 26900 Lodi, Italy; francescabernini96@libero.it (F.B.); alessandro.bagnato@unimi.it (A.B.); stefano.marelli@unimi.it (S.P.M.); luisa.zaniboni@unimi.it (L.Z.); silvia.cerolini@unimi.it (S.C.)

**Keywords:** autochthonous breeds, SNPs, turkey, genomic inbreeding, ROH, population genetic diversity

## Abstract

Italian autochthonous turkey breeds are an important reservoir of genetic biodiversity that should be maintained with an in vivo approach. The aim of this study, part of the TuBAvI national project on biodiversity, was to use run of homozygosity (ROH), together with others statistical approaches (e.g., Wright’s F-statistics, principal component analysis, ADMIXTURE analysis), to investigate the genomic diversity in several heritage turkey breeds. We performed a genome-wide characterization of ROH-rich regions in seven autochthonous turkey breeds, i.e., Brianzolo (Brzl), Bronzato Comune Italiano (BrCI), Bronzato dei Colli Euganei (CoEu), Parma e Piacenza (PrPc), Nero d’Italia (NeIt), Ermellinato di Rovigo (ErRo) and Romagnolo (Roma). ROHs were detected based on a 650K SNP genotyping. ROH_islands were identified as homozygous ROH regions shared by at least 75% of birds (within breed). Annotation of genes was performed with DAVID. The admixture analyses revealed that six breeds are unique populations while the Roma breed consists in an admixture of founder populations. Effective population size estimated on genomic data shows a numeric contraction. ROH_islands harbour genes that may be interesting for target selection in commercial populations also. Among them the *PTGS2* and *PLA2G4A* genes on chr10 were related to reproduction efficiency. This is the first study mapping genetic variation in autochthonous turkey populations. Breeds were genetically different among them, with the Roma breed proving to be a mixture of the other breeds. The ROH_islands identified harboured genes peculiar to the selection that occurred in heritage breeds. Finally, this study releases previously undisclosed information on existing genetic variation in the turkey species.

## 1. Introduction

According to historical evidence, turkey domestication originated in Mexico and Central America. After the Spanish conquerors brought the turkeys to Europe, the novelty and the appreciated meat characteristics permitted a rapid spread across Europe, starting from the 16th century. Since their diffusion in Europe, turkey populations were then bred divergently in the next centuries [1]. In the last 40 years, the turkey species underwent a targeted selection for meat production in order to obtain a fast-growing, heavy bird; nowadays several hybrids, born out of an intensive selection program, are used in intensive farming to produce turkey meat.

The Italian autochthonous turkey populations show a wide phenotypic variation among breeds, spanning plumage colour, body size and weight [2]. Heritage breeds can be considered an in situ genetic variability reservoir that should be maintained and their value exploited. The heritage populations, in fact, own unique characteristics making them especially valuable for their capability to adapt to harsh environments and to resist diseases [3]. Their reduced body size with respect to commercial hybrids, additionally, is particularly suited for local traditional Italian cuisine.

Since these turkey breeds are reared in rural and family farms, the size of the populations is at present very limited. To date, many actions have already been taken to preserve the turkey’s biodiversity. The Italian Ministry of Agriculture (MIPAAF) established the Registro Anagrafico delle Razze Avicole Autoctone (RAA; Autochthonous Italian Poultry Breeds Registry) (DM 19536, 1 October 2014). Furthermore, the European Agricultural Fund for Rural Development funded the TuBAvI project “Tutela della Biodiversità nelle razze AVicole Italiane”. The aim of this project was to safeguard, preserve and improve the Italian poultry genetic resources, which are represented by all the autochthonous breeds. This project has been granted a follow-up, the TuBAvI-2, broadening and consolidating the knowledge on Italian breed populations gained with TuBAvI.

Costs to obtain SNP high-throughput genotypes have decreased in recent years, favouring the production of genomic data and the application of genomic analyses in livestock populations. This occurred either where genomic selection (e.g., bovine breeds) has been adopted or in small and endangered populations where in situ genetic conservation plans are desired. Heritage turkey breeds were recently studied and, even if still limited, some indication about their genomic variability and diversity is nowadays available [4,5,6,7,8,9]. Recent studies were performed on run of homozygosity (ROH): Marras et al. characterized ROH-rich regions in a commercial turkey hybrid, and Strillacci et al. discussed the difference in ROH between the autochthonous Mexican and commercial hybrid turkeys [10,11].

ROHs are continuous genomic regions defined by adjacent markers in a homozygous state that are identical by descent as a result of processes such as inbreeding, population size reduction and natural selection [12]. ROH’s frequency and length can be used to understand the inbreeding level and the populations’ genetic history [13]. In addition, ROH can be used to identify how far from present the inbreeding occurred in the mating events of the populations: in fact, long ROHs identify recent inbreeding whereas a short ROH indicates a more ancient one [14]. The homozygous segments may also have an important impact on the expression of complex traits for production traits as well as for diseases [15,16,17].

In this study, we analysed the genetic variability among seven Italian turkey breeds registered in the RAA (Brianzolo, Bronzato Comune, Bronzato dei Colli Euganei, Parma e Piacenza, Nero d’Italia, Ermellinato di Rovigo and Romagnolo) and we performed a genome-wide characterization of ROH-rich regions in order to identify the genome tracts under recent and ancient inbreeding as determined by ROH.

## 2. Materials and Methods

### 2.1. Sampling and Genotyping

In this study, 181 birds’ genotypes from the Axiom^®^ TurkeyHD Genotyping Array of seven Italian turkey breeds (Brianzolo: Brzl n. 31, Bronzato Comune: BrCI n. 24, Colle Euganei: CoEu n. 22, Parma e Piacenza: PrPc n. 25, Nero d’Italia: NeIt n. 26, Ermellinato di Rovigo: ErRo n. 24, and Romagnolo: Roma n. 29) were available from Strillacci et al. (Brzl, NeIt, CoEu, and 12 samples of PrPc) and from Strillacci et al. (BrCI, ErRo, Roma, and 13 samples of PrPc) [8,9]. The two genotype datasets were appended and underwent a joint quality control.

Filtering was applied to retain for the analyses SNPs with genome position on autosome (first 30 chrs) and having a call rate >99%.

A total of 346,155 SNP markers passed the quality control, and their location on the genome was in concordance with the Turkey_5.0 genome assembly—GCA_000146605.1.

### 2.2. Population Genetic Diversity Analyses

The genetic diversity within and among breeds was determined using the following approaches:

- The number of monomorphic SNPs, the expected and observed heterozygosity (Exp Het and Obs Het), the expected and observed number of homozygous SNPs (Exp Hom and Exp Hom) and minor allele frequency for each breed were calculated.
- A principal components analysis (PCA) was performed based on allele genotypes using the SNP & Variation Suite (SVS) v8.9 (Golden Helix Inc., Bozeman, MT, USA). The graphical visualization of PCA was obtained by the ggplot2 R package [18].
- The pairwise fixation index (i.e., Wright’s F-statistic F_ST_) was estimated using the dedicated module implemented in SVS. The F_ST_ was estimated for all possible pairs of breed combinations.
- Identity-by-state estimates of genetic distances were calculated for all pairs of individuals using PLINK 1.9. A neighbour-joining tree (NJ) was constructed—using the distance matrix as input file—by Phylip software implemented in the online WebPHYLIP tools [19]. A NJ tree was then drawn with MEGA X software [20].
- The ADMIXTURE analysis: The most probable number of ancestral populations was identified in conjunction with the lowest cross-validation error (CV), setting the analysis with optimal number of clusters (K-value) from 2 to 8. PLINK 1.9 software [21] was used to generate the input file to run ADMIXTURE analysis [22].
- Effective population size (Ne) for each breed was predicted using the SNeP_111 software [23] based on linkage disequilibrium (LD).

### 2.3. Identification of Runs of Homozygosity (ROHs)

ROHs were computed using the consecutive run method implemented in SVS 8.9 software. We restricted ROH segments to longer than 1 Mb to avoid identification of ROH resulting from LD. In addition, a minimum of 150 homozygous SNPs was required to be in each run, and no heterozygote or missing SNPs were allowed in the ROH, and the maximum gap between SNPs was set to 1000 Kb.

The mean number, the average length of ROH, the average sum of ROH segments per breed, as well as the ROHs distribution into four classes of length (1–2, 2–4, 4–8 and 8–16 Mb) were estimated per breed.

The genomic regions most commonly associated with ROH (ROH_islands) were defined within breed as clusters of runs found in more than 75% of samples using a specific option implemented in SVS software during the ROH detection: samples within the same cluster had identical ROH (length, position on genome and boundaries).

### 2.4. Gene Annotation of ROH_Islands and Functional Analyses

The completed list of turkey genes annotated according to the most recent reference genome was downloaded from NCBI online Database (NCBI Annotation Release: 103). Genes with an official gene name and “LOC” ID associated both with a protein coding and official gene name were catalogued within the identified ROH_islands using the “intersectBed” command of BEDTools software [24]. The Database for Annotation, Visualization and Integrated Discovery (DAVID), v6.8, was interrogated to perform a gene ontology (GO) functional annotation and KEGG pathway analyses.

Additionally, considering that to date no quantitative trait loci (QTLs) database is available for turkeys, the one accessible for the chicken species, i.e., the Chicken QTLdb—based on Galgal 6.0 genome assembly, was used to identify—using the “Search by associated gene” option—QTLs overlapping the genes found in the ROH_islands.

### 2.5. Inbreeding Coefficients

Two genomic inbreeding coefficients for all the birds were estimated based on:

- F_HOM_—the difference between the observed and expected numbers of homozygous genotypes within the population calculated using the routine implemented within SVS;
- F_ROH_—the ratio calculated between the total length (sum) of all ROH of an individual and the total length of the autosomal genome covered by the SNP marker dataset, 902,020,024 bp in this study.

## 3. Results

### 3.1. Breed Diversity among All Breeds

The proportion of monomorphic SNPs was variable according to breed, spanning from 21% (NeIt) to 77% in ErRo (Table 1).

The observed (Obs Het) and expected (Exp Het) heterozygosity values were in concordance with the monomorphic SNPs and with minor allele frequency (MAF) values ranging from 0.06 (ErRo) to 0.20 (PrPc).

Table 1 also reports the observed (Obs Hom) and expected (Exp Hom) number of homozygous SNPs for all turkey breeds analysed and, as observable, the highest values were identified in the ErRo breed showing a proportion of 92.0% of Obs Hom.

**Figure 1:**
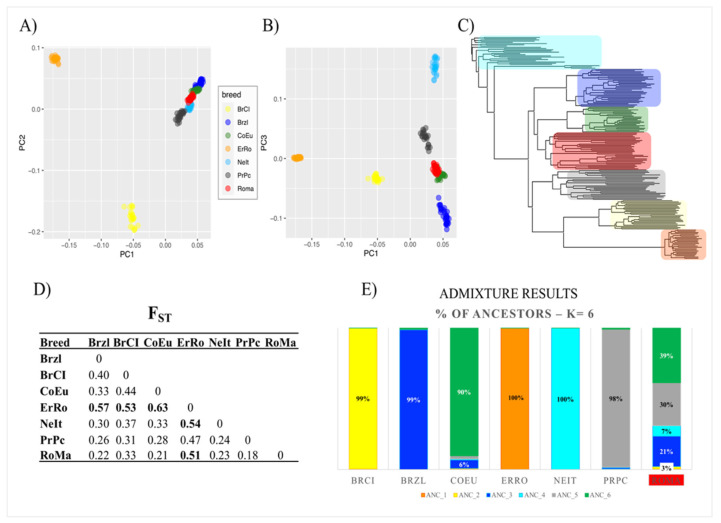
Breeds’ genetic diversity based on SNP markers. (**A**) PCA 2D result: PC1 (eigenvalues = 18.70; 29.02% of total genetic variability) vs. PC2 (eigenvalues = 10.41; 16.16% of total genetic variability); (**B**) PCA 2D result: PC1 vs. PC3 (eigenvalues = 8.44; 13.09% of total genetic variability); (**C**) NJ tree built using the identity-by-state genetic distance matrix (breeds are coloured as in PCA); (**D**) FST results: in bold FST values >0.5; (**E**) ADMIXTURE result: proportion (more relevant values are reported) of ancestors identified (“ANC_n.”) at K = 6, where the lowest CV error value was found.

The principal component analysis (PCA) results are shown in the graphs of Figure 1A,B: (i) the first two principal components (PC1 eigenvalue of 18.94–29% of total variation; PC2 eigenvalue of 8.44–16.2% of total variation) allow to clearly distinguish ErRo and BrCI breeds from the other ones clustered in the adjacent space; (ii) PC3 separates the remaining breeds, in particular NeIt birds and Brzl are well separated from PrPc, CoEu and Roma birds. Birds belonging to CoEu and Roma breeds appear to be two very close groups, showing partial overlapping.

Comparable results were found by NJ tree analysis (Figure 1C) and by the pairwise breed comparisons (F_ST_ values) (Figure 1D), confirming that ErRo is the more distant breed from the others with an F_ST_ value >0.47 for all comparisons).

The ADMIXTURE analysis revealed that at K = 6 (where the lowest CV error value was identified), six breeds seem to be mostly unique populations, with an ancestral genetic composition defined by a proportion of 90% in CoEu and equal (PrPc) or larger than 98% (Brzl, BrCI, ErRo, NeIt). The Roma breed appeared to have a composite ancestral genetic contribution from CoEu (39%), PrPc (30%), NeIt (7%), Brzl (21%) and BrCI (3%) (Figure 1E).

**Figure 2:**
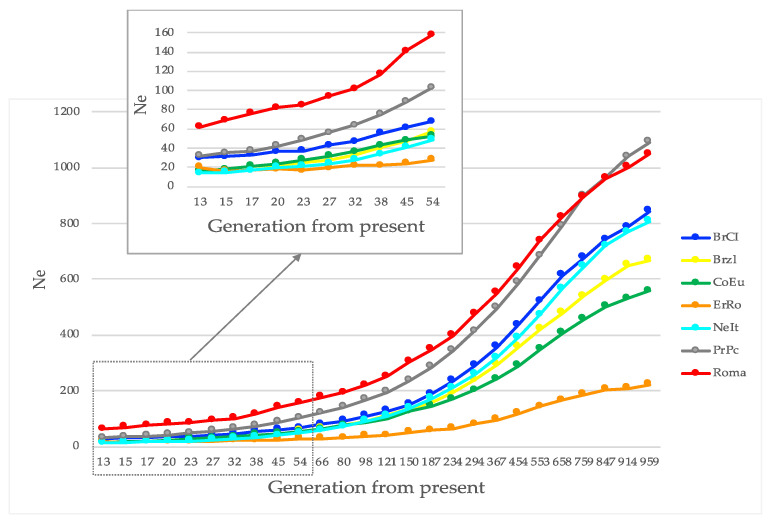
Effective population size (Ne) calculated up to about 950 past generations for each breed.

Figure 2 shows the effective population size trends calculated for each breed up to 950 past generations.

### 3.2. Run of Homozygosity

ROHs were identified in all birds of each breed for a total of 20,858 homozygous regions (Table S1). The largest average number of ROHs per animal was observed for the CoEu breed (156.2), while the lower ones were identified in PrPc (62.8) and Roma (69.3) (Table 2). The longer average ROH lengths were observed for Brzl and CoEu breeds (about 2 Mbps) while the lowest were found for the BrCI and Roma breeds (about 1.7 Mbps). The portion of the genome covered by ROH is more than two times larger in ErRo and CoEu compared to the ones for NeIt, PrPc and Roma.

**Figure 3:**
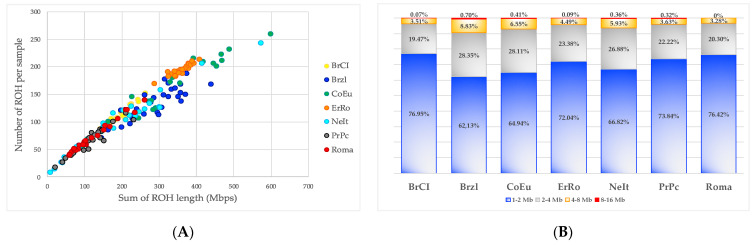
Breeds’ diversity based on ROH. (**A**) Relationship between the number and mean length of ROH (Mb = Mega bases); (**B**) proportion of ROH for each class of length calculated within each breed.

The graphical representations of ROH statistics by breeds are shown in Figure 3. In detail, (i) Figure 3A represents the relationship between the number and the mean total length of ROHs for each individual according to breed; (ii) Figure 3B shows the proportion of ROH classified by taking into account the length. As shown, the ROH < 2Mb class of length is the most represented for all seven breeds, whereas the 4–8 Mb and the 8–16 Mb classes of length are the less frequent ones. The Roma breed did not have ROHs longer than 8 Mb.

### 3.3. Incidences of Common Runs per SNP and ROH_Island Identification

In order to explore how the mating plans in each breed have affected their genetic structure, the ROH_island regions were considered and analysed. Using the SNP incidences, the ROH_islands were defined as a genomic homozygous region with (i) the same boundaries (and as such the same chromosome length) and (ii) shared in at least 75% of samples of a given breed (threshold values are a function of each breed’s sample size).

**Figure 4:**
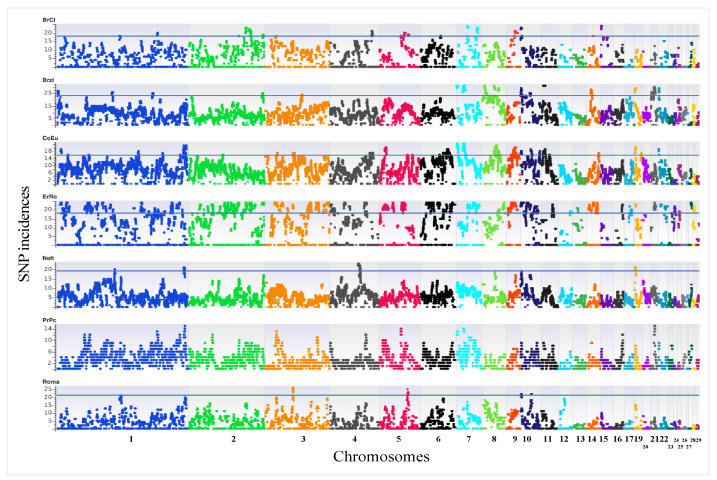
Manhattan plots of SNP incidences for all turkey breeds.

As shown in Figure 4, the genomic distribution of SNP incidence defining ROH_islands is clearly non-uniform across autosomes in all breeds. Table S2 reports the ROH_islands together with the genes annotated in each of them and the overlapping QTLs that were identified interrogating the Chicken QTLdb. A total of 130, 21, 17, 15, 3 and 1 ROH_islands were found in ErRo, Brzl, BrCl, CoEu, NeIt and Roma, respectively. No ROH_islands were identified for the PrPc breed.

**Figure 5:**
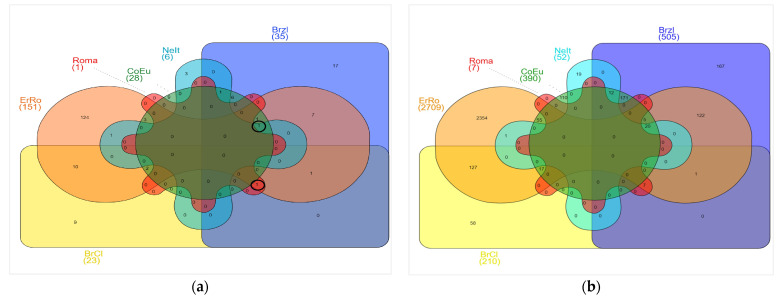
Venn diagram of proper (specific of one breed) and common ROH_islands (shared by at least two breeds) (**a**) and annotated genes (**b**). Circled frequencies of common ROH_island regions are those shared by four breeds.

A different proportion of proper (identified only in one breed) and common ROH_islands (shared by at least two breeds) were observed among the populations (Figure 5a,b). Two ROH_islands were shared by four breeds (BrCI, Brzl, ErRo, Roma—chr 10; Brzl, CoEu, ErRo, NeIt—chr 19) (Table 3). A total of 25 other ROH_islands were common among breeds with various combinations.

Table 3 reports the list of ROH_islands identified in more than one breed, together with annotated genes and the overlapping QTLs. The functional annotation of genes annotated in the ROH_islands (*p*-value <0.05) is reported in Table S3.

**Figure 6:**
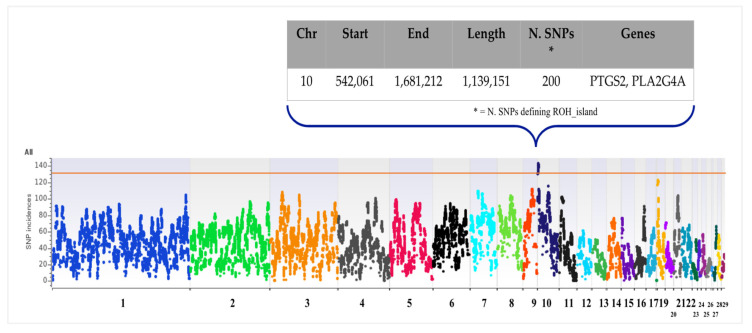
Manhattan plots of SNP incidences defining the ROH_island identified in overall birds, together with annotated gene and associated traits.

When we considered all birds as a unique population, an ROH_island on chr10 was identified (Figure 6) in at least 75% of turkeys (>n. 136, out of a total of 181 birds). This analysis had the goal of finding a genomic homozygous region shared among all breeds as a possible result of common selection for traits related to survival of the species, such as reproductive efficiency.

Among the 200 SNPs defining this ROH_island in all 136 samples (same boundaries in all of them), 18 SNPs were mapped in the intronic position of the *PTGS2* (n.2) and *PLA2G4A* (n.16) genes, the only ones annotated in this genomic region. Table S4 reports the allele counts for each of these markers calculated per breed: (i) in all ErRo birds, the homozygous allele was always the alternative one, compared to homozygous birds in other breeds for all these 18 SNPs, without any heterozygous SNPs: e.g., if ErRo turkeys were homozygous AA, all the homozygous birds of Brzl, BrCI, CoEU, NeIt, and Roma were BB. For all these breeds, the individuals with heterozygous SNPs were negligible as shown in Table S4. In the PrPc samples, the distribution of genotypes for this region was about 25% AA, 50% AB and 25% BB.

### 3.4. Parma e Piacenza ROH_Islands

Although no ROH_islands for the PrPc breed were found at the threshold considered (i.e., 75% of animals of a breed having a specific ROH), two homozygous regions were shared by a large proportion of individuals, 60%, corresponding to 15 birds. These two regions mapped on chr 1 (at 185,658,695–185,769,482) and on chr 21 (at 5,328,921–5,922,971) (Table 3) and harboured a total of 18 genes (Table S5).

### 3.5. Inbreeding Coefficients

**Figure 7:**
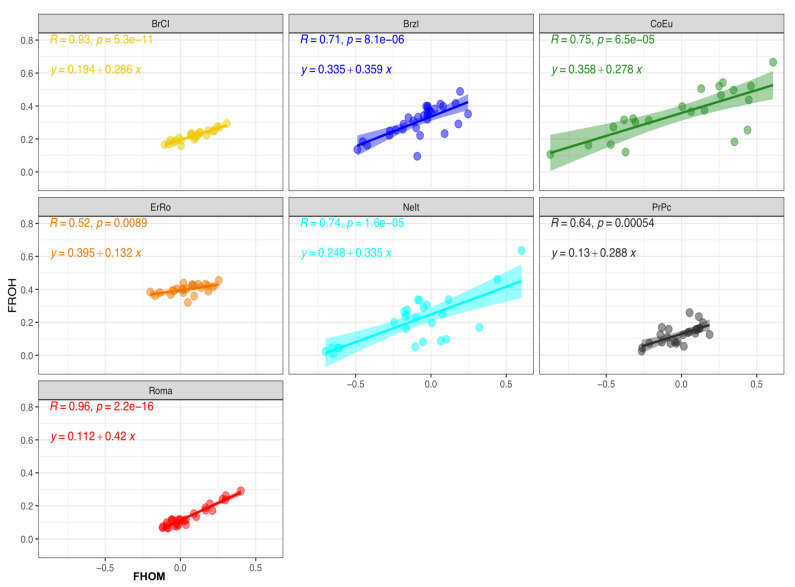
Linear regression and correlation values together with descriptive statistics for F_HOM_ and F_ROH_ (Table 4) in each turkey breed.

Two genomic inbreeding coefficients F_HOM_ and F_ROH_ have been calculated and are reported in Table 4. Figure 7 shows the linear regression and correlation values estimated in each turkey breed. Slightly negative F_HOM_ mean values were calculated for CoEu, NeIt, and PrPc while positive close to 0 ones were found for the remaining breeds. CoEu birds showed the largest variability in F_HOM_ values.

As the F_ROH_ values were calculated based on ROH proportion over the genome length, they reflect the ROH distribution in each sample, its average length across the classes and the total genome length covered by ROH (Figure S2). Differences in F_ROH_ were also found across the chromosomes of all breeds (Figure S3). Linear regressions between the two inbreeding values (F_HOM_ and F_ROH_) obtained for each breed are reported in Figure 7 together with correlation coefficients (i.e., R in each graph of Figure 7). Correlation values were found to have a medium value, i.e., from 0.52 to 0.75 in Brzl, CoEu, ErRo and NeIt, to a strong one, i.e., 0.93 in BrCI and 0.96 Roma.

## 4. Discussion

### 4.1. Genetic Diversity of Breeds

Autochthonous turkey breeds have undergone to a population size contraction after the diffusion of meat type hybrids, more efficient in growth performance and carcass yield. Nowadays, heritage turkey breeds represent a unique genetic biodiversity reservoir. Their in situ conservation is particularly important as the autochthonous breeds own unique characteristics, including adaptability to extensive farming in harsh environments. Their characterization, as envisaged in the TuBAvI project, allows the identification of the phenotypic and the genetic characteristics related to their intrinsic value, especially if compared to selected hybrids that, even though very efficient in meat production performances, may exhibit suboptimal performance in other traits, e.g., reproduction efficiency [17,25].

This study showed that the genetic composition of each of the seven autochthonous turkey breeds differs from others. Based on the PCA and the NJ tree analyses, birds of each breed clustered together revealing a consistent genetic composition. The ErRo and BrCI are the two more genetically distant breeds from the other ones and, according to the F_ST_ values, the ErRo is the one more differentiated from all the other ones: the F_ST_ values were 0.47 and 0.63 when ErRo was compared with PrPc and CoEu, respectively. A possible explanation is that the feather colour of this breed, white with black streaks, clearly different from plumage colour of other breeds, has been chosen as a selection criterion by the breeders and farmers causing, as such, a divergent selection with respect to the other breeds. The pairs Roma–PrPc (F_ST_ = 0.18) and Roma–CoEu were less differentiated (F_ST_ = 0.21). The relative closeness of Roma and PrPc could be related to the geographic origin of these breeds, the northern area of Italy below the Po river in the Emilia-Romagna region (eastern part i.e., Romagna). The Roma breed standard is not as stringent as the one of the other breeds, in fact it does not include a specific trait such as the feather colour as in the ErRo. This may have facilitated the cross of birds of the breeds geographically closer to the Romagna region, i.e., Veneto (CoEu), Emilia (PrPc) and Lombardia (Brzl), but not the ErRo due to its colour. It is interesting to note that the proportion of ancestors found in the Roma breed using the ADMIXTURE analysis was inversely proportional to the geographical distance of Veneto, Emilia and Lombardia from Romagna. With the exclusion of the Roma breed, the ADMIXTURE analysis revealed for all the other ones the uniqueness of the genetic background for each of the Italian breeds with a proportion of each ancestor >90%.

### 4.2. ROH and ROH Islands

In the last decades, the in situ maintenance, i.e., the reproduction of Italian autochthonous turkeys, was mostly carried on by private breeders. They had two main selection goals: (i) to maintain the morphological characteristics of each breed; (ii) to improve birds’ performance in a semi-extensive farming system.

The meat of these breeds is mainly used for traditional cuisine recipes for private consumption; the backyard farming as a consequence is not centred on growth performance but on meat quality (i.e., muscle fibre consistency for long cooking) and resilience of birds to pastoral extensive farming.

Generally, the reproductive scheme used by private breeders is avoiding as much as possible increases in inbreeding, adopting as strategy of toms’ exchange between farms. Although the selection in autochthonous breeds is not as focused and intense as the one in other livestock species in pure breeding, it determined consequences in the genome makeup of the turkey breeds, including the formation of ROH_islands.

The population size of each breed is particularly small because its contraction occurred in the last 4 decades: assuming a generation interval of 1 year (a realistic value due to the seasonality of reproduction in autochthonous breeds), the graph in Figure 2 shows the decrease of Ne in the last 45 generations for each breed.

It is interesting to note that the Ne of the ErRo was the smallest among all, with a fairly constant value in the last 30 generations. This is a further evidence supporting the closeness of the nucleus of the ErRo population in the last decades. The Roma breed, in accordance with the ADMIXTURE results, exhibited the largest Ne possibly because of the inclusion of birds from different breeds.

The population size of these breed is very small as reported by the FAO DAD-IS database and by Castillo et al. ranging from 9 (PrPc) to 445 (BrCI) birds across the breeds and all considered at critical risk of extinction [26,27]. Due to the very limited population size, the total number of homozygous SNPs was then found to be quite high in all the breeds, with the higher proportion in the ErRo, where the total number of homozygous SNPs was 318,213 (92% of the total SNP dataset). As discussed hereinbefore, the ErRo breeders aim at a strict maintenance of the morphological standard of the feather colour, limiting the number of reproducers and the variability existing in the breed. This might be one of the reasons determining a higher homozygosis in this breed with respect to the other ones having a proportion of homozygous loci ranging from 72% to 84%. The ErRo also had the highest proportion of SNPs in ROH_islands suggesting, together with other results here presented, that the selection for the specific colour of plumage might have played a role in building a larger proportion of ROH_islands. The ErRo, in particular, is the only breed showing a clear homozygosity state and ROH_island for all individuals (see Supplementary Materials, Figure S4) on chr1: 41,354,392–41,408,938 that harbours the *KITLG* gene, associated with melanin patterning [28] and involved in skin coloration in vertebrates [29]. Additionally, the ErRo had the highest number of ROH_Islands, 130 vs. 21, 17, 15, 3 and 1 found in Brzl, BrCI, CoEu, NeIt and Roma, respectively. However, the proportion of ROH_Islands overlapping with those identified in other breeds was only 17% (mainly with BrCI, n. 12), suggesting that a large proportion of regions is under specific selection pressure in the ErRo.

The Roma breed is the one with the smallest proportion of homozygous SNPs in ROH. Additionally, there are no ROHs longer than 8 Mb. This result agrees with the ADMIXTURE one, indicating a possible introgression in the breed from various ones as hereinbefore supposed.

A similar high level of homozygosity has been identified in Italian local chicken breeds [30], where low values of heterozygosity underline the difficulties of maintaining a fair level of genetic variability in breeds under an in situ conservation plan.

As shown in Table 3, 27 ROH_Islands were shared at least by two breeds. Two ROH_Islands were shared by four populations (Table 3): (i) on chr 10—BrCI, Brzl, ErRo and Roma; (ii) on chr 19—Brzl, CoEu, ErRo and NeIt.

The ROH_Island located on chr10 is shared by the highest number of birds across breeds, i.e., n. 155 (n. 136 turkeys is considered the threshold of 75% of samples), as shown in Figure 5. In this ROH_Island, 200 consecutive SNPs were homozygous in all ErRo and in the major part of turkeys belonging to the other breeds; 18 homozygous SNPs mapped in the intronic position of the *PRGS2* and *PLA2G4A* genes (Figure 6).

To assess the genetic variability existing in this species, it is important to characterize the heritage breeds’ specific genetic biodiversity, especially in comparison to the highly selected heavy turkey lines. Comparing the evidence of the ROH_Islands in this region to existing data on ROH in commercial hybrids, it is interesting to note that the *PTGS2* and *PLA2G4A* genes were found by several authors to be related to reproductive physiology in avian and other livestock species [31,32,33,34,35,36]. The wide occurrence of this ROH in a large number of animals of autochthonous turkey populations under an outbreeding reproduction scheme suggests that the genes included in this region may be under selection because they are important for reproduction and thus for the survival of the species. NeIt and CoEu are two breeds that in the recent past were appreciated and bred for their good egg production. Except the Roma, the PrPc and one bird of CoEu (heterozygous), all other birds were in a homozygous state in and in the proximity of the two genes here mentioned. The ErRo was in a homozygous state but with the alternate allele with respect to the other breeds (i.e., Brzl, BrCI, NeIt, CoEu) as shown in Supplementary Materials, Table S4. Comparing the results of this study with the ROHs mapped in a commercial turkey hybrid, this region does not appear to be in a homozygous state in the commercial selected, fast-growing, heavy turkey line as reported by Strillacci et al. [11]. The intense selection for body weight in commercial turkeys determined a reduction in reproductive performance, as reported by Nestor et al., who discussed the genetics of growth and reproduction using the performance measured in over 40 generations of selection for 16-week body weight in turkey [37]. Interestingly, the PrPc, having the largest proportion of heterozygous individuals in this region (i.e., f(A) = f(B) = 0.50), is also the heaviest of all the Italian breeds [8,9] reaching an adult weight doubling the one of the other breeds (about 12 kg in males vs. 5–7 kg of other breeds; about 6.5 kg in females vs. 3–4 kg in other breeds). At present, the studies on genomic structure in turkeys are very limited as also recently pointed out by Adams et al. [38] and, to best of our knowledge, no other published data are available on ROHs in hybrid turkeys or in selected pure lines.

The second region mapped on chr19 in four breeds harboured genes connected to heat-stress-related functions (*TRAF2*) [39] and involved in hypoxia adaptation in high altitude (*NOXA1*) [40], meat quality traits [41], feeding behaviour (relation to nutrient stress) and mating (with relation to survival behaviour) (*GRIN1*) [42].

#### Comparison with Literature

To the best of our knowledge, only three studies [10,11,38] performed a genome-wide ROH detection in local (Mexican), hybrid and pure-line turkey populations. Among the ROHs detected by these authors, only those found by Strillacci et al. are available online: among these published ROHs, only two overlapped with those here identified (in Brzl breed), both on chr 8 at 4,344,134–5,525,301 and at 5,536,601–6,787,697 [11]. In these two regions, 15 genes are annotated, some of which are related to meat juiciness and tenderness (*MMRN2* [43,44]), fat cell development and fatty acid metabolism (*ADIRF* [45]), and circadian rhythms (*Opn4* [46]).

### 4.3. Inbreeding Coefficients

The possibility of calculating genomic inbreeding values is useful in the absence of pedigree information, a very common condition in avian species. This is particularly important in local and endangered populations such as autochthonous Italian turkey breeds. In our study, populations showed genomic inbreeding with a large variability of mean values as shown in Table 4: F_HOM_ mean values range from −0.118 to 0.141 while F_ROH_ mean values vary from 0.126 to 0.401. The variability of inbreeding values within breeds is also very different across them: considering F_ROH_, the SD of the mean values ranges from 0.035 to 0.153 showing that the effect of selection and the management of reproductive practices breeders are influencing in a variable manner the genetic makeup of these populations.

The two breeds with lower value of F_ROH_ are the Roma and the PrPc, reflecting the reproductive structure and management of the two populations. The Roma breed is an admixture of different turkey breeds as hereinbefore commented. The PrPc is, together with the Roma, the breed with largest Ne as reported in the graph of Figure 2. Additionally, the observed heterozygosity in the PrPc is the highest among all. The size reduction of the populations during the last decades is for sure having an impact on the genomic inbreeding value: the number of farms and the frequency of exchange of birds among breeders may also affect inbreeding values. Unfortunately, up to 2014, with the setup of the RAA, there is no track of the reproducers used in the populations, making the genomic approach a unique resource for mapping the existing variability among breeds.

A positive correlation was found between F_HOM_ and F_ROH_ in all breeds, with R values ranging from moderate to strong (>0.90 in BrCI and Roma). These results, attributable to a higher proportion (from 62% to 77%) of short ROHs in all breeds, are consistent with those reported in the literature on livestock [11,47,48,49] and confirm the helpfulness of ROHs in the estimation of the inbreeding coefficient, mainly in small populations where pedigree information is difficult to collect or not available.

## 5. Conclusions

Information on the genetic variability of turkey breeds is very limited and, to the best of our knowledge, this is the first study on autochthonous turkey populations. The wide diffusion of commercial, fast-growing, heavy hybrids in the last 40 years has impacted the farming of autochthonous populations, affecting in the same manner the existing genetic variability in the turkey species. The wide spread of commercial hybrids has in fact reduced the farming of heritage breeds, causing a loss of genetic variation and biodiversity. Local breeds are well adapted and selected over centuries to deal with environmental harsh conditions of semi-extensive farming system. The knowledge of their genomic variability is indeed an important step forward with respect to the state of the art, today mainly based on selected heavy turkeys. This study is in fact part of the nation-funded project TuBAvI that aims to investigate the existing biodiversity in local avian populations using genomic data and to provide insights for their in situ conservation. This study releases previously unavailable information on the genomic variation existing in autochthonous turkey populations, showing regions under selection. The results of this project represent as such a unique resource to compare the genetic variability of autochthonous turkey breeds with the one of selected commercial turkeys. The use of ROHs to exploit genomic variation in autochthonous populations revealed genomic regions under selection (ROH_islands) that harbour genes potentially interesting also for a targeted selection in commercial lines. The results here obtained are, as such, a unique resource in mapping genomic variation in turkey species.

## Figures and Tables

**Table 1:** Breeds’ descriptive statistics for monomorphic SNP count and proportion (%), observed (Obs Het) and expected (Exp Het) heterozygosity, minor allele frequencies (MAF), observed (Obs Hom) and expected (Exp Hom) number of homozygous SNPs, and number of Hom SNPs defining ROH and outside of ROH.

Breeds	No. of Birds	No. of SNPs	Monomorphic SNPs ^1^	Obs Het ^2^	Exp Het ^2^	MAF	No. of Obs Hom SNPs (%) ^1^	No. of Exp Hom SNPs	No. of Hom SNPs in ROH (%) ^3^	No. of Hom SNPs Outside ROH (%) ^3^
BrCI	24	346,068	148,532 (42%)	0.178	0.194	0.14	284,335 (82.2%)	278,233	64,700 (23%)	219,634 (77%)
Brzl	31	345,913	150,568 (43%)	0.213	0.195	0.15	272,329 (78.7%)	277,398	94,956 (35%)	177,373 (65%)
CoEu	22	345,799	128,032 (37%)	0.163	0.158	0.11	289,382 (83.7%)	289,937	106,955 (37%)	182,426 (63%)
ErRo	24	346035	265,019 (77%)	0.080	0.082	0.06	318,213 (92.0%)	316,920	117,437 (37%)	200,775 (63%)
NeIt	26	345,648	71,610 (21%)	0.248	0.221	0.17	258,660 (75.2%)	267,855	63,383 (25%)	195,276 (75%)
PrPc	25	345,932	91,406 (26%)	0.275	0.265	0.20	250,984 (72.5%)	252,303	37,842 (15%)	213,142 (84%)
Roma	29	346,091	97,122 (28%)	0.223	0.230	0.17	268,984 (77.7%)	264,978	36,650 (14%)	232,334 (86%)

^1^ Proportion of total number of SNPs; ^2^ mean value; **^3^** proportion of homozygous SNPs.

**Table 2:** Runs of homozygosity (ROHs) descriptive statistics; ROH lengths are expressed in base pairs (bps).

Breed	Total ROH	Min ^1^ ROH	Max ^2^ ROH	Average ROH no. (SD)	Min ^1^ ROH Length	Max ^2^ ROH Length	Mean ROH Length	Mean Coverage (%) ^3^
BrCI	2707	86	151	112.8 (17.3)	1,001,226	9,639,308	1,758,260	198,317,129 (21.98)
Brzl	4000	57	187	129 (33.5)	1,000,237	14,291,629	2,158,213	278,479,051 (30.87)
CoEu	3437	54	259	156.2 (58.6)	1,000,666	12,307,208	2,048,563	320,041,453 (35.48)
ErRo	4657	169	213	194 (10.3)	1,001,605	10,680,210	1,862,868	361,474,000 (40.07)
NeIt	2477	8	242	95.3 (57.4)	1,001,182	10,341,755	1,973,087	187,974,437 (20.83)
PrPc	1570	17	115	62.8 (25)	1,000,040	14,195,544	1,807,656	113,520,780 (12.58)
RoMa	2010	38	139	69.3 (27.8)	1,000,469	6,949,958	1,726,671	119,676,128 (13.26)

^1^ Min: minimum; ^2^ Max: maximum; ^3^ mean length covered by ROH (proportion of genome—defined by the SNP dataset—covered by ROH).

**Table 3:** ROH_islands found in common among breeds together with the annotated genes (NCBI Annotation Release: 103—Turkey), and the overlapping QTLs (reference QTL_IDs refer to chicken studies—Chicken QTLdb). ROH lengths are expressed in base pairs (bps).

Breed	Chr	Start	End	Genes	QTL
CoEu, ErRo	1	5,531,217	7,117,134	*SEMA3D, GRM3, KIAA1324L, DMTF1, TMEM243, MEIG1, DCLRE1C, SUV39H2, HSPA14*	
Brzl, ErRo	1	81,065,017	81,284,840	*CCDC191, ZDHHC23, GRAMD1C, CLDND1, GPR15, ILDR1, CFAP44, BOC*	
BrCI, ErRo	2	72,136,043	73,206,329	*EPHA7*	
BrCI, ErRo	2	82,001,208	83,935,025	*ADGRB3, SMARCD3, CHPF2, ABCF2, ASB10, GBX1, AGAP3*	
BrCI, ErRo	2	87,210,702	89,080,482	*LRRC1, MLIP, TINAG, FAM83B, HCRTR2, HMGCLL1, GFRAL, DST, BEND6, ZNF451, RAB23, BAG2*	
Brzl, ErRo	2	106,019,973	107,545,271	*OPN5, ADGRF4, TNFRSF21, ANKRD66, MEP1A, ADGRF5, IMP3, TDRD6, PLA2G7, RCAN2, ENPP4, CLIC5, RUNX2, SUPT3H, MUT, RHAG, CENPQ*	RUNX2: Body weight (21 days) (QTL:137385); Feed intake (QTL:137384); Femur weight (QTL:137388); Growth (35–41 days) (QTL:138385); Lung weight (QTL:137387); Wing weight (QTL:137386);
CoEu, ErRo	5	7,242,173	10,024,527	*TRIM66, ST5, RPL27A, TMEM9B, ASCL3, C5H11orf16, AKIP1, NRIP3, SCUBE2, DENND5A, ZNF143, WEE1, SWAP70, AMPD3, RNF141, MRVI1, LYVE1, EIF4G2, GALNT18, GALNTL4, DKK3, USP47, MICAL2, PARVA, TEAD1, RASSF10, PTH, BTBD10, ARNTL, OVCH2, PPFIBP2*	PTH: Eggshell percentage (QTL:12860); Eggshell strength (QTL:12861)
BrCI, ErRo	5	34,641,163	37,301,923	*EGLN3, EAPP, SNX6, CFL2, BAZ1A, SRP54, FAM177A1, PPP2R3C, KIAA0391, PSMA6, NFKBIA, RALGAPA1, BRMS1L, MBIP, NKX2-1, NKX2-8, SLC25A21, MIPOL1, FOXA1, TTC6, SSTR1, SEC23A, GEMIN2, TRAPPC6B, PNN, MIA2, FBXO33, ZNF410, FAM161B, COQ6, ENTPD5, BBOF1, ALDH6A1, LIN52, VSX2, ABCD4, VRTN, SYNDIG1L, ISCA2, NPC2, LTBP2, AREL1, FCF1, YLPM1, PROX2, DLST, RPS6KL1, PGF, EIF2B2, MLH3, ACYP1, ZC2HC1C, NEK9, TMED10*	EGLN3: Feather pecking (QTL 15660); RALGAPA1: Age at first egg (QTL:17157; 17165; 17155; 17161; 17153; 17159), Egg number (QTL:17164; 17160; 17152; 17158; 17156); MIPOL1: Feather pigmentation (QTL:137129)
Brzl, CoEu	7	2684	1,851,174	*SLC38A11, ADARB1, FAM207A, ITGB2, KMO, C7H21orf58, PCNT, DIP2A, S100B, LSS, POFUT2, MCM3AP, FTCD, COL6A2, COL6A1, PCBP3, SLC19A1, COL18A1, NDUFA10, HDAC4, TWIST2, ASB1, TRAF3IP1, USP40, SH3BP4, ARL4C, SPP2, TRPM8, AGAP1*	ASB1: Body slope length (QTL:106165; 106154; 106166); Body weight (28 days) (QTL:106160); Body weight (56 days) (QTL:106161); Body weight (84 days) (QTL:106162); Body weight (hatch) (QTL:106153; 106159); Breast bone crest length (QTL:106164); Breast muscle weight (QTL:106167); Carcass weight (QTL:106156; 106168; 106155; 106158); Drumstick and thigh muscle weight (QTL:106157); Shank length (QTL:106163). TRPM8: Eggshell color (QTL:193596)
BrCI, ErRo	7	15,781,590	16,965,319	*GYPC, BIN1, ERCC3, MAP3K2, PECR, XRCC5, TMEM169, SMARCAL1, RPL37A, IGFBP2*	
BrCI, ErRo	7	29,839,072	31,129,536	*LYPD6B, KIF5C, EPC2, MBD5, ACVR2A, ORC4*	
Brzl, CoEu	8	17,575,286	18,772,065	*FRMPD2, MAPK8, ARHGAP22, WDFY4, LRRC18, VSTM4, C8H10orf128, C8H10orf71, DRGX, ERCC6, CXCL12, TMEM72, ANKRD22, STAMBPL1, ACTA2, FAS, CH25H, LIPA, SLC16A12*	CXCL12: Alternative complement activation by BRBC (QTL:14582; QTL:14584)
Brzl, CoEu, ErRo	8	23,527,407	24,302,723	*COL17A1, SFR1, ITPRIP, CFAP58, SORCS3*	
ErRo, CoEu	9	9,522,827	11,249,410	*CLCN5, SLITRK4, GABRQ, GABRA3, GABRE, CNGA2, PRRG3, RIPPLY1, CLDN2, TMLHE, SPRY3, VAMP7, ZNF185, NSDHL, CETN2*	
BrCI, CoEu, ErRo	9	11,695,727	12,737,859	*ARHGEF9, CDX4, CHIC1, RLIM, KIAA2022, ABCB7, UPRT, ZDHHC15, FGF16, ATRX*	
BrCI, CoEu, ErRo	9	12,768,468	13,273,536	*MAGT1, ATP7A, PGK1, AMOT, TRPC5, ALG13, DCX, CAPN6, PAK3*	
BrCl, ErRo	9	13,273,537	14,162,682	*CHRDL1, AMMECR1, TMEM164, ACSL4, NXT2, IRS4, COL4A5, COL4A6, ATG4A, PSMD10, VSIG1, RAB39B, VBP1, GAB3*	
BrCl, ErRo	9	14,166,574	15,370,901	*GAB3, SMARCA1, TENM1, SH2D1A*	
Brzl, ErRo, BrCI, Roma	10	311,121	1,681,212	*HMCN1, PRG4, TPR, C10H1orf27, PDC, PTGS2, PLA2G4A*	
Brzl, ErRo	10	13,391,015	14,421,595	*TNR, KIAA0040, TNN, MRPS14, GPR52, RABGAP1L, CACYBP, ZBTB37, SERPINC1, DHX9, NPL, LAMC1, NMNAT2, LAMC2, ARPC5, NCF2, SMG7, APOBEC4, RGL1, COLGALT2, C10H1orf21, ZNF326, LRRC8D, LRRC8C*	
Brzl, CoEu	11	2,817,886	5,515,380	*C11H3orf58, SLC9A9, CHST2, U2SURP, PAQR9, TRPC1, PCOLCE2, PLS1, ATR, XRN1, TFDP2, ATP1B3, GRK7, RASA2, ZBTB38, SLC16A14, TRIP12, PID1, SPHKAP, DAW1, CCL20, AGFG1, MRPL44, MFF, COL4A3, COL4A4, RHBDD1, IRS1, NYAP2*	SLC9A9: Triglyceride level (QTL:165716)
Brzl, ErRo	13	6,306,916	7,466,233	*CNEP1R1, HEATR3, ADCY7, BRD7, NKD1, SNX20, CYLD, SALL1*	
BrCI, ErRo	13	8,804,570	9,987,519	*MMP2, LPCAT2, CAPNS2, SLC6A2, SLC12A4, DDX28, DUS2, NFATC3, ESRP2, PLA2G15, SMPD3, PRMT7, SLC7A6, SLC7A6OS*	
CoEu, Brzl, ErRo, NeIt	19	34,288	1,233,014	*PRF1, CCDC189, ANKMY1, DPP7, MAN1B1, UAP1L1, SAPCD2, ENTPD2, TMEM141, C19H9orf142, CLIC3, ABCA2, FUT7, NPDC1, C8G, FBXW5, TRAF2, EDF1, MAMDC4, PHPT1, C19H9orf172, RABL6, AMBP, EXD3, NOXA1, TMEM203, NDOR1, RNF208, RNF224, LRRC26, GRIN1, ZNF618*	
Brzl, ErRo, PrPc*	21	4,202,233	6,435,093	*AUTS2, TYW1, SEPT4, CCDC183, SBDS, LIMK1, ELN, CLDN3, ABHD11, MTMR4, HSF5, RNF43, STX1A, WBSCR22, MKS1, BCL7B, TBL2, MLXIPL, BAZ1B, FZD9, **POM121C, NSUN5, HIP1, LPO, CCL26, CCL24, SCYA4, TSPOAP1, SUPT4H1, PITPNM3, FAM64A, FBXO39, TEKT1, SMTNL2, KIAA0753, MED31, TXNDC17, SLC13A5, XAF1, WSCD1**, RAP1GAP2, PAFAH1B1, CLUH, MNT, SGSM2, TNFAIP1, TMEM97, IFT20, NLK, KSR1, NOS2, WSB1*	SGSM2: Body weight (112 days) (QTL:65719); NLK: Body weight (112 days) (QTL:65723; 65728); NOS2: Cecal bacterial burden after challenge with Salmonella E (QTL:13647)
Brzl, ErRo	21	6,443,945	6,881,999	*NF1, EVI2A, EVI2B, OMG, AKAP1, CUEDC1, MRPS23, VEZF1, DYNLL2, HEATR6*	NF1: Body weight (112 days) (QTL:65726)
Brzl, CoEu	22	83,111	1,297,642	*L3MBTL1, SRSF6, EPB41L1, DLGAP4, TGIF2, MYL9, C22H20orf24, GGT7, NCOA6, TP53INP2, PIGU, TLDC2, SAMHD1, RBL1, CHD6, RALGAPB, NDRG3, SLA2, PHF20, RBM39, ROMO1, NFS1, CPNE1, RBM12, ERGIC3, CEP250, GDF5*	

* PrPc: ROH_island identified in 60% of birds: the annotated genes are in bold. Underlined genes are those associated with phenotypes (QTL).

**Table 4:** Descriptive statistics for inbreeding coefficients F_HOM_ and F_ROH_.

Breed	F_HOM_			F_ROH_		
	Min	Max	Mean (SD)	Min	Max	Mean (SD)
BrCI	−0.105	0.31	0.089 (0.115)	0.159	0.293	0.220 (0.035)
Brzl	−0.49	0.25	−0.074 (0.18)	0.095	0.488	0.308 (0.091)
CoEu	−0.871	0.61	−0.009 (0.411)	0.106	0.666	0.355 (0.153)
ErRo	−0.2	0.25	0.044 (0.118)	0.321	0.455	0.401 (0.03)
NeIt	−0.7	0.6	−0.118 (0.32)	0.011	0.636	0.208 (0.148)
PrPc	−0.264	0.19	−0.014 (0.127)	0.025	0.259	0.126 (0.057)
Roma	−0.119	0.4	0.049 (0.144)	0.067	0.291	0.133 (0.063)

## Data Availability

All data here produced are included in the additional files.

## Supplementary Materials

The following are available online at https://www.mdpi.com/article/10.3390/genes12091342/s1, Figure S1: Box plot of F_HOM_ values calculated for each breed, Figure S2: Box plot of F_ROH_ values for each class of ROH length; Figure S3: F_ROH_ values calculated for each chromosome; Figure S4: ROH_island on chr1 containing *KITGL* gene: homozygosity genotypes for all ErRo’s birds. Table S1: List of ROH identified for all turkeys; Table S2: List of ROH_Islands per breed; Table S3: Annotation of genes performed using DAVID database; Table S4: SNPs mapped in *PTGS2* and *PLA2G4A* genes and turkey genotypes; Table S5: ROH_Islands identified in Parma e Piacenza (PrPc) breed.
