# Supplementary material for: Genetic Diversity and Identification of Homozygosity-Rich Genomic Regions in Seven Italian Heritage Turkey (Meleagris gallopavo) Breeds
Source: Genes (Basel). 2021 Aug 28;12(9):1342. doi: 10.3390/genes12091342 (PMC8470100; doi:10.3390/genes12091342)

Identification of homozygosity-rich genomic regions in seven Italian local turkey (*Meleagris gallopavo*) breeds

Figure S1. Box plot of FHOM values calculated for each breed.

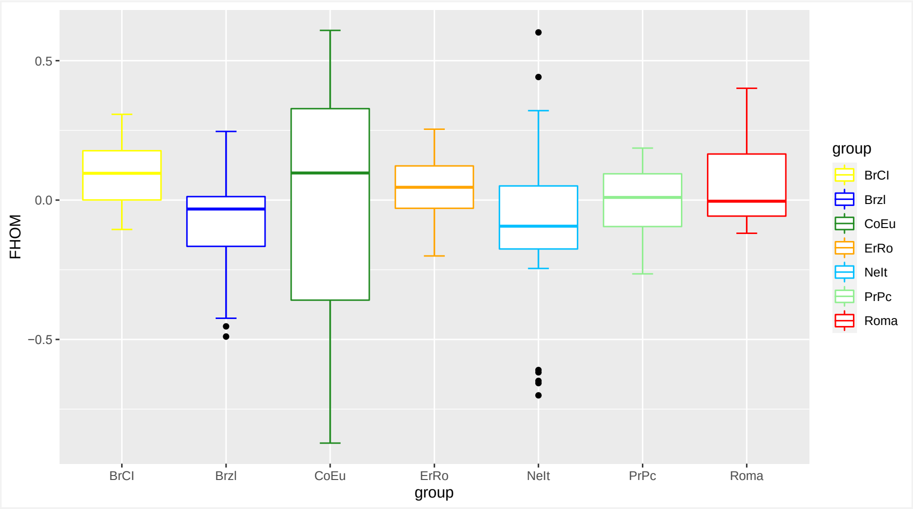

Figure S2. Box plot of FROH values for each class of ROH length

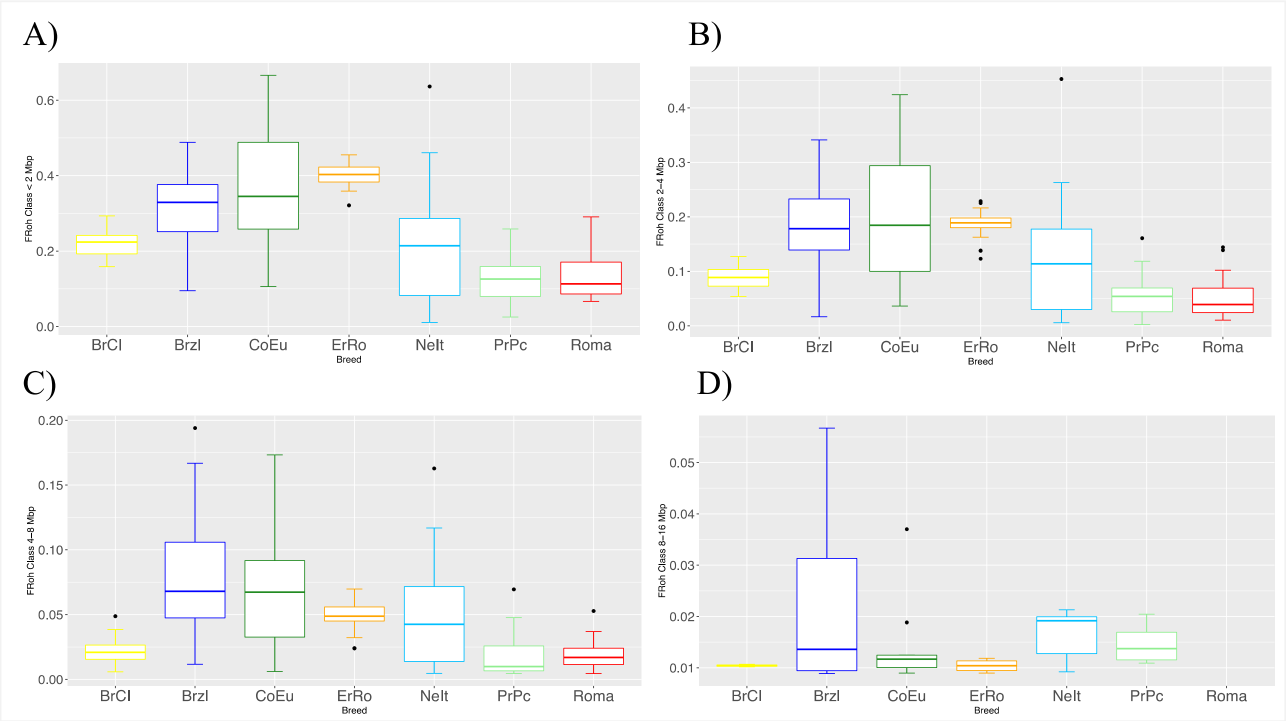

Figure S3. F_{ROH} values calculated for each chromosome.

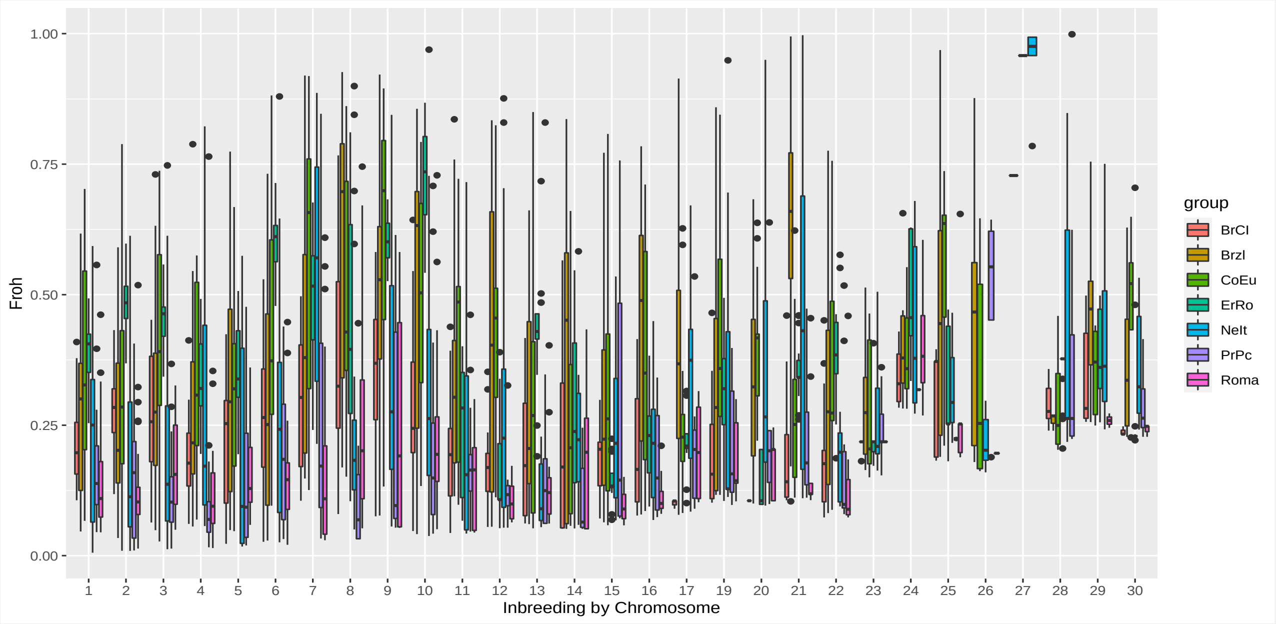

Figure S4. ROH_island on chr1 containing KITGL gene: homozygosity genotypes for all ErRo's birds.

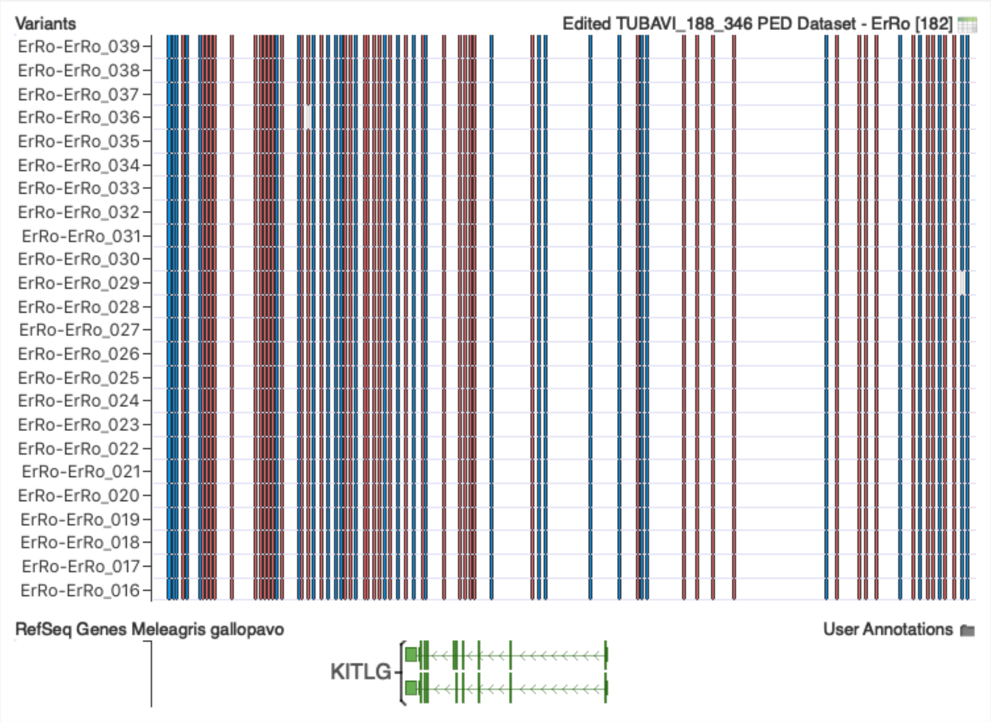

Supplement: Supplementary file 1 [file genes-12-01342-s001.zip › Additional_Figures.pdf]
